# Interactions between parasites and microbial communities in the human gut

**DOI:** 10.3389/fcimb.2012.00141

**Published:** 2012-11-16

**Authors:** Federica Berrilli, David Di Cave, Serena Cavallero, Stefano D'Amelio

**Affiliations:** ^1^Department of Experimental Medicine and Surgery, Tor Vergata UniversityRome, Italy; ^2^Department of Public Health and Infectious Diseases, Sapienza UniversityRome, Italy

**Keywords:** parasites, protozoans, helminths, microbiota, parasitome, pathogenesis, immune system, probiotics

## Abstract

The interactions between intestinal microbiota, immune system, and pathogens describe the human gut as a complex ecosystem, where all components play a relevant role in modulating each other and in the maintenance of homeostasis. The balance among the gut microbiota and the human body appear to be crucial for health maintenance. Intestinal parasites, both protozoans and helminths, interact with the microbial community modifying the balance between host and commensal microbiota. On the other hand, gut microbiota represents a relevant factor that may strongly interfere with the pathophysiology of the infections. In addition to the function that gut commensal microbiota may have in the processes that determine the survival and the outcome of many parasitic infections, including the production of nutritive macromolecules, also probiotics can play an important role in reducing the pathogenicity of many parasites. On these bases, there is a growing interest in explaining the rationale on the possible interactions between the microbiota, immune response, inflammatory processes, and intestinal parasites.

## The human intestinal microbiota

The human gut represents a complex ecosystem composed by a large microbial community associated with the human body (Human Microbiome Project Consortium, 2012). The species composition varies greatly between individuals, with each individual harboring a unique collection of bacterial species, which may change over time (Bäckhed et al., 2005; Eckburg et al., 2005; Qin et al., 2010). Genetic factors play an important role in gut microbiota development, although environment also drives species acquisition (Zoetendal et al., 2001). Recently, the human body together with its gut microbiota has been referred to as a “superorganism” where an extensive coordination of metabolic and physiological processes occurs (Nicholson et al., 2004). The presence of the intestinal microbiota enriches the human organism with important functions, particularly in regulating host fat storage (Bäckhed et al., 2004), stimulating intestinal epithelium renewal (Rakoff-Nahoum et al., 2004), and influencing the maturation of the immune system (Mazmanian et al., 2005).

As recently reviewed (Sekirov et al., 2010; Clemente et al., 2012), the balance among the gut microbiota and the human body is crucial for health maintenance, and perturbation of microbial composition has been supposed to be involved in a range of diseases (Bäckhed et al., 2005; Palming et al., 2006). Moreover, the commensal microbiota contributes to the “barrier effect” of the intestinal epithelium, which plays the primary role of protecting the host, representing a real obstacle to pathogens invasion (Bancroft et al., 2012). Within this complex scenario, intestinal parasites interact with the microbial community modifying the balance between host and gut microbiota. Each of these organisms metabolizes and modifies substrates interactively. Resident microbiota products may strongly interfere with the survival and the physiology of many parasites and, consequently, with the outcome of many parasitic infections. On the other hand, intestinal parasites, both protozoans and helminths, constantly excrete and secrete molecules that may change the environment determining alterations in gut microbiota compositions. Also part of the energy extracted from nutrient metabolism by resident microbes may be beneficial not only to the host (Sekirov et al., 2010) but also to parasitic organisms eventually present. It is therefore pertinent to consider the intestinal environment as an ecosystem where biological and chemical interactions occur at various organizational levels between host, parasites, and microbial communities (Nicholson et al., 2004; Bancroft et al., 2012).

## Protozoans

A wide range of protozoans are common parasites of human gastro-intestinal tract. They are a not homogenous group and their physiology and biochemistry are largely geared to the parasitic habit. They show different mechanisms of host invasion, some are intracellular (e.g., *Cryptosporidium* spp.) and host specialized (e.g., *Entamoeba histolytica*), many of them are adapted to more than one host (e.g., *Giardia duodenalis*). Few species do any real damage but some occasionally give rise to symptoms that usually include diarrhea related to damage in the wall of the bowel.

Among protozoans, the species *G. duodenalis* could represent a good model to highlight some mechanisms related to the existing interactions with the intestinal microbiota. This flagellate is recognized as one of the most common pathogenic gastrointestinal parasites in humans and in a wide range of animals (Thompson, 2000). The spectrum of clinical manifestations varies from a mild self-limiting illness to acute or chronic diarrhea and weight loss, with malabsorption lasting for several months (Farthing, 1996). Furthermore, people may be infected without any symptoms. The causes determining this variability in clinical picture are still poorly understood.

Numerous studies assessed that pathogenesis results from interaction between parasite products, such as proteinases that break the epithelial barrier, and host inflammatory and immunological responses as observed for *Cryptosporidium* (Chai et al., 1999; Guk et al., 2003) as well as for *Giardia* (Scott et al., 2004; Ankarklev et al., 2010). Recognition of protozoans parasitizing mucosal surfaces may involve innate immune system, e.g., toll-like receptors (TLRs), as demonstrated *in vivo* on infected humans for *Trichomonas vaginalis* (Zariffard et al., 2004), and *in vitro* on human monocytes for *E. histolytica* (Maldonado et al., 2000). Moreover, T cells (particularly involving CD8+ cells), macrophages, neutrophiles, and antibodies (IgM, IgG, and IgA) are major players of the acquired immune response necessary for the resolution of giardiasis.

### Protozoan infection and gut microbiota

Gut microbiota represents an additional factor that may strongly interfere with the pathophysiology of the parasite infections. However, the existing interactions between the enteric flora and protozoan parasites are still poorly understood.

Based on mouse models, normal intestinal flora was shown to decrease susceptibility to infection by *Cryptosporidium parvum* (Harp et al., 1992). Conversely, in other studies, the presence of gut microbiota seems to be essential for the pathogenic expression of other enteric protozoans such as *E. histolytica* (Phillips et al., 1955), *Blastocystis hominis* (Phillips and Zierdt, 1976), and different species of *Eimeria* (Visco and Burns, 1972; Owen, 1975; Gouet et al., 1984).

Different hypotheses have been proposed to explain the mechanisms involved in this pathogenic stimulation by bacteria. Some of them are related to changes caused by axenisation of the protozoans. In this case, the surface saccharide ligands of the superficial membrane are altered by the presence of intracellular bacterial symbionts, so that in axenic protozoa cured of their endosymbionts, a possible decrease in adhesion or in invasive abilities can be observed (Phillips, 1973; Dwyer and Chang, 1976). Also in *Giardia*, in the past decade, ultrastructural observations of *Giardia muris* in a murine model revealed endosymbiotic microbes which, according to the authors, could be related to variation in the trophozoite pathogenicity, metabolism, range of infectivity, antigenic surface characteristics, and host specificity (Nemanic et al., 1979). More recently, the presence of *Giardia* trophozoites harboring peripheral bacterial endosymbionts was also demonstrated by El-Shewy and Eid (2005). Based on TEM examination, the authors found that only trophozoites with endosymbionts were lysed when in close vicinity of the activated Paneth cells, confirming the host protective role of the bacterial endosymbionts within *Giardia* trophozoites and further supporting the idea that gut microbiota may directly and indirectly interfere in the pathogenesis of giardiasis.

Similarly intriguing is the idea that axenisation of the host at the intestinal level can be involved in the virulence expression of protozoan parasites. Working with *E. histolytica*, Mirelman and colleagues (1982, 1983) evidenced that interactions of amoebae of low pathogenicity with a variety of Gram-negative bacteria, mainly *Escherichia coli* strains, may be responsible for the increase in amoebic virulence. More recently, Galván-Moroyoqui et al. (2008) demonstrated that phagocytosis of enteropathogenic bacteria strains (e.g., *E. coli* and *Shigella dysenteriae*) *in vitro* co-cultured with *E. histolytica* and *Entamoeba dispar* augmented the cytopathic effect of *E. histolytica* and increased expression of Gal/GalNAc lectin on the amoebic surface and the cysteine proteinase activity. *E. dispar* remained avirulent.

Also for *G. duodenalis*, several studies have shown that the intestinal microbiota can stimulate the pathogenic expression but not the multiplication of parasites (Torres et al., 1992, 2000). In a gnotobiotic animal model, Torres et al. (2000) provided evidence that the bacteria responsible for part of the stimulation of *G. duodenalis* pathogenicity are present in the dominant duodenal microbiota. In this work, facultative and strictly anaerobic micro-organisms of the duodenal microbiota were obtained from biopsy of five children with symptomatic giardiasis and tested for their ability to stimulate *G. duodenalis* pathogenicity in gnotoxenic mice. Quantification of cysts in faeces and of trophozoites in the small bowel was also performed to evaluate protozoan multiplication in the different groups of mice. As observed, germ-free animals did not develop intestinal pathological modifications during experimental *Giardia* infection; infected gnotoxenic mice showed intermediate pathological alterations between germ-free and infected conventional mice used as controls; finally, no pathological changes were observed in non-infected gnotoxenic or conventional animals. According to the authors, these results support the hypothesis that, as demonstrated also for other intestinal pathogenic protozoans, bacterial components from the intestinal microbiota represent stimulatory factors for *Giardia* pathogenicity but not for protozoan multiplication since faecal cyst levels remained similar among the three different groups of mice during the experimental infection.

## Helminths

The intestine represents the ideal habitat for a large number of parasitic worms. Among flatworms, cestodes of the genera *Diphyllobothrium, Taenia*, and *Hymenolepis* and digeneans such as *Fasciolopsis, Heterophyes*, and *Schistosoma*, live in close interaction with human gut mucosae and lumen. As for nematodes, the most common intestinal roundworms are geohelminths (*Ascaris, Trichuris*, Ancylostomatidae, and *Strongyloides*), as well as *Enterobius vermicularis*.

While in less-favored areas, the interest in intestinal helminthiases is mainly focused on the parasitic disease itself, in industrialized countries the intimate relationships between intestinal helminthes with gut microbiota and the putative down-regulation of self-pathogenic immune response have been the object of recent studies, as a consequence of the increasing concern regarding childhood allergies, atopic dermatitis and asthma (Patel et al., 2008), IBDs like Crohn's disease and ulcerative colitis, and autoimmune disorders (Weinstock and Elliott, 2009).

### Helminth infection and gut microbiota

The human intestinal microbiota is essential in providing nourishment, regulating epithelial development, and instructing innate immunity (Eckburg et al., 2005). A significant variability and differences between community compositions are often described, all consistent with a picture of a highly diverse ecosystem. It has been suggested that, in the course of helminth infections, significant changes in the abundance and composition of gastrointestinal tract microbiota are observed. Intestinal nematodes produce molecules that may alter the habitat for gut microbiota. Walk et al. (2010) showed that infection of mice with *Heligmosomoides polygyrus*, a parasite of the duodenum, induces changes in composition of bacteria communities in the ileum but not in the colon; the majority of bacteria within the infected ileum were Lactobacillae species. Interestingly, *H. polygyrus* is able to significantly reduce inflammation of colitis in mice (Elliott et al., 2004) and to determine alterations in epithelial barrier function in the colon (Su et al., 2011). Additionally, Li et al. (2012) demonstrated a significant alteration in the colon microbiota of pigs induced by *Trichuris suis* after 21 days from infection. As suggested by Wu et al. (2012), the initial infection, even when not followed by the persistence of the parasitosis, is able to determine changes in the abundance of up to the 13% of genera detected, in particular *Fibrobacter* and *Ruminococcus.*

A further major aspect related to the helminth infections is the potential interaction between macrofauna, microflora, and host immunity. It has been evidenced the overall decrease in proinflammatory cytokines associated with chronic inflammation observed in the course of helminth infections; moreover, autoimmune disorders have a reduced incidence in geographical regions where higher prevalence of parasitic infections are reported (Sewell et al., 2002). Reddy (2010) argued that a reduced exposure to pathogenic organisms in developed countries may determine a minor stimulation of the immune system and an increased incidence of autoimmune and allergic diseases in the human populations. Based on several studies from developing country settings, evidences have been provided for the role of intestinal nematodes in the prevention of allergic responses (van den Biggelaar et al., 2004; Summers et al., 2005a; Croese et al., 2006; Leonardi-Bee et al., 2006; Flohr et al., 2009). This phenomenon is known as “Hygiene hypothesis” (Wills-Karp et al., 2001; Weinstock and Elliott, 2009). In particular, the interactions between helminth infections and host immune system may prove to be beneficial for both, the parasite and the host, with regard to the control of autoimmune diseases (Maizels et al., 2009).

On this basis, there is a growing interest in explaining the rationale on the existing interactions between helminthes, gut microbiota and immune-mediated intestinal inflammatory status, e.g., in celiac patients, as recently reviewed by Bancroft et al. (2012). The authors, focusing mainly on infections due to *Trichuris* sp., considered the immunomodulation by parasitic helminths and the interaction between the microbiota and the immune system in an integrated manner, where Th17 (T-helper) and Tregs (regulatory T cells) are affected by the action of microbiota, and are in turn able to act on parasite survival. At the same time, parasitic worms produce molecules that may alter the habitat for intestinal microbiota.

Besides to nematodes, also digeneans such as *Schistosoma mansoni* have been described to induce microbial disturbance. In a metabonomic investigations in mice infected with *S. mansoni*, Wang et al. (2004) reported several complex outcomes to the metabolism disturbance due to *Schistosoma* infections, including impaired liver functions, perturbation of amino acids metabolism and of the tricarboxylic acid (TCA) cycle. Moreover, high excretion of urinary trimethylamine, phenylacetylglycine, and p-cresol glucuronide indicating disturbances in the gut microbiota are found in *S. mansoni*-infected mice, probably due to an increased production from microbial agents caused by alteration of the microbial ecosystem in the presence of the parasite. Analogous changes at the Nuclear Magnetic Resonance (NMR) metabolic profiles have been detected during the infections by *Fasciola hepatica, Necator americanus*, and other human helminth parasites, as reviewed by Wang et al. (2010).

Similarly, Balog et al. (2011), on the base of urinary response of rodent and human hosts to *S. mansoni* infection, demonstrated gross disturbance of metabolites associated with gut microbial community and microbial co-metabolism and Li et al. (2011) identified 12 urinary and five faecal metabolites as biomarkers of *Schistosoma* infection, able to differentiate infected and not infected mice, adding further evidence to the hypothesis that *S. mansoni* infection either directly or indirectly modulates host gut microbial activity.

### Nematodes as a therapy

The positive results in the potential of therapeutic effect of worms or their molecules in animals have led to several human studies exploring presumptively harmless helminthes like *T. suis*, a whipworm that naturally infects pigs. Treatment of colitis patients with *T. suis* ova provided promising results and such therapies are currently under development (Summers et al., 2005b). However, a special attention should be paid to possible adverse effects. The first concern regards the zoonotic potential of *T. suis*. The systematics of the group is still controversial and a clearcut delineation of species infecting humans is actually under definition. The second aspect is related to the possibility that *T. suis* infection may play a role in the internalization of intestinal pig epithelial cells by bacteria (e.g., *Campylobacter jejuni*) and subsequent bacterial invasion (Wu et al., 2012). Finally, the effect of helminth infections on allergic diseases may vary depending on the parasite species, as it has been proposed that different species may act as immunosuppressant or as enhancers of allergic phenomena (Pinelli, 2012).

## Probiotics against parasites

Probiotics may also be a factor that can potentially inhibit the development of several pathogens. As reviewed by Travers et al. (2011), probiotics demonstrated to be efficient for the treatment of gastrointestinal disorders, respiratory infections, and allergic symptoms, and also can kill or inhibit pathogens by strain-specific mechanisms relying on competition, molecule secretion, and/or immune induction. Several studies have reported the effects of probiotics on parasites, both protozoans (e.g., *Cryptosporidium*, *Eimeria*) and helminths (e.g., *Ascaris*, *Trichuris*).

As regard *Giardia*, a large amount of data are now available, since the first study of Singer and Nash (2000) who provided preliminary evidences that the composition of the intestinal flora was likely involved in the highly variable manifestations in giardiasis in both humans and animals. Pérez et al. (2001) studied the *in vitro* effect of different probiotic bacteria (six *Lactobacillus acidophilus* strains, and *Lactobacillus johnsonii* La1) on *G. duodenalis* strain WB trophozoites demonstrating that only *L. johnsonii* La1 significantly inhibited the proliferation of *Giardia* trophozoites. The activity of *L. johnsonii* La1 (NCC533) was confirmed by Humen et al. (2005) in *in vivo* experiments where a protection against parasite-induced mucosal damage and a cellular response to *Giardia* antigens was stimulated in spleen cells from La1-treated animals, leading to a resolution of infection.

Moreover, *Lactobacillus casei* MTCC 1423 strain as well as *Enterococcus faecium* SF68 were both effective in eliminating *Giardia* infection in probiotic-fed mice by minimizing or preventing the adherence of *Giardia* trophozoites to the mucosal surface (Shukla et al., 2008) and stimulating an humoral response (Benyacoub et al., 2005).

Recently, the effectiveness of different lactobacilli species/strains to prevent and treat murine *Giardia* infection has been further assessed by several authors (Shukla et al., 2009, 2010; Goyal et al., 2011). The results obtained by Shukla and Sidhu (2011) and Shukla et al. (2012) showing the positive effect of *L. casei* in renourished *Giardia intestinalis* infected BALB/c mice confirm the role of probiotics to reduce the duration and severity of giardiasis through the morphological and physiological retrieval of the intestine.

As for worms, Bautista-Garfias et al. (2001) suggested that oral treatment with *L. casei* appears to reduce the parasite burden *Trichinella spiralis* in mice. Also *Enterococcus faecalis* CECT7121, a probiotic with inhibitory activity against Gram-positive and Gram-negative bacteria, possesses *in vitro* and *in vivo* larvicidal activity determining up to a 90% reduction of the number of *Toxocara canis* larvae in liver and lungs of laboratory mice (Basualdo et al., 2007; Chiodo et al., 2010). *Zymomonas mobilis*, a bacterium producer of bioethanol, was reported to provide over 60% protection from the infection of *S. mansoni* in mice (Santos Jde et al., 2004).

## Future perspectives

The multidimensional linkages among human body, gut microbiota and parasites result in a complex ecosystem where alterations in one of the these components determine a counter response in the remaining ones. In this view, in order to achieve an advanced understanding of the ongoing processes determining the infections, an -omics approach which include comprehensive, multidisciplinary and combined actions from these different perspectives is needed. Many outstanding questions require further investigations, e.g., the existing interactions between the microbiota, immune response, inflammatory processes, and intestinal parasitic diseases as well as the mechanisms regarding how probiotics act against intestinal parasites and the possibility for their therapeutic use for humans. Finally, new exciting areas such as the study of the parasitome and the metabolome of gut microbiota during chronic parasite infection and their relationship with host immunoregulatory mechanisms have now to be approached in the framework of an integrated approach.

### Conflict of interest statement

The authors declare that the research was conducted in the absence of any commercial or financial relationships that could be construed as a potential conflict of interest.
